# Role of Ion Channels in the Pathophysiology of SARS-CoV-2

**DOI:** 10.3390/v18091027

**Published:** 2026-09-16

**Authors:** Faisal F. Alousi, Abdel A. Alli

**Affiliations:** 1 Department of Medicine, Division of Nephrology, Hypertension, and Renal Transplantation, University of Florida, College of Medicine, Gainesville, FL 32610, USA; f.alousi@ufl.edu; 2 Department of Physiology and Aging, University of Florida, College of Medicine, Gainesville, FL 32610, USA; 3 Department of Physiology, Abdulrahman Bin Faisal University, College of Medicine, Dammam 31441, Saudi Arabia

**Keywords:** SARS-CoV-2, viroporins, ion channels

## Abstract

In the pathophysiology of SARS-CoV-2, the critical role of ion channels has become increasingly evident during the COVID-19 crisis. This review offers a comprehensive analysis of the functions of ion channels, including viroporins, in both the viral life cycle and the host immune response. It explores how ion channels facilitate viral replication, assembly, and immune evasion. Moreover, this review critically examines the molecular mechanisms underpinning SARS-CoV-2 infection, with a particular emphasis on the integral role of viral proteins exhibiting membrane-interacting properties, collectively named viroporins, in promoting viral survival and triggering host defense mechanisms, highlighting their potential as therapeutic targets. By unraveling these complex interactions, this review aims to increase our understanding of COVID-19 pathophysiology.

## 1. Introduction

The COVID-19 pandemic, caused by severe acute respiratory syndrome coronavirus 2 (SARS-CoV-2), posed significant global health challenges. We focus here on one part of SARS-CoV-2 biology, membrane-active viral proteins called viroporins, because they appear to play an important role in viral replication, cell stress, and disease severity.

SARS-CoV-2 entry into host cells begins with the viral Spike (S) protein binding to the angiotensin-converting enzyme 2 (ACE2) receptor [1] and in some instances, the neuropilin-1 (NRP1) receptor on the host cell surface [2]. This receptor binding facilitates viral attachment, followed by proteolytic cleavage of the Spike protein by host cell proteases such as Transmembrane Protease, Serine 2 (TMPRSS2), furin, or cathepsins. This cleavage is a critical step, as it primes the Spike protein, enabling it to facilitate the fusion of the viral envelope with the host cell membrane, thus initiating viral entry [3]. Another phenomenon of entry is called apoptotic mimicry, where the phosphatidylserine (PS) receptors, that are normally used to clear dying cells, are utilized by SARS-CoV-2. Exposed PS is an ‘eat me’ signal and is readily detectable on the surface of virions. This route can be considered as secondary, since Spike-ACE2 binding remains the primary mechanism [4].

Once inside the host cell, the viral RNA genome is released into the cytoplasm, where it hijacks the host cell machinery to translate and replicate its RNA. This process involves the synthesis of viral replicative/non-structural proteins, which form the replication-transcription complex that drives the replication and transcription of the viral genome [5]. Among these proteins are the RNA-dependent RNA polymerase Non-Structural Protein 12 (nsp12) responsible for RNA synthesis, and proteases such as nsp3 and nsp5, which cleave the viral polyprotein into functional units [6].

One important part of the viral replication strategy involves viroporins, which are encoded by SARS-CoV-2 to manipulate the host cell environment. The term ‘viroporin,’ first introduced by Nieva et al., refers to a class of typically small, predominantly hydrophobic viral proteins possessing the capacity to form transmembrane pores and alter membrane permeability, a subject extensively reviewed [7]. Ion channels are membrane proteins that facilitate the passage of ions across cell membranes and are crucial for numerous physiological processes [8,9,10]. In this review, we provide a distinction between viroporins and bona fide ion channels. A viroporin alters membrane permeability while an ion channel is characteristic for having selective ion movement, measurable conductance, and functional behavior consistent with gating. Viral ion channels therefore fall within the broader class of viroporins, but not all viroporins show the selectivity or gating behavior of canonical ion channels. During viral infections, these viroporins, whether acting as general pore-forming or specific channels, manipulate host cell environments to favor viral replication and dissemination [11]. SARS-CoV-2 encodes such proteins, including the Envelope (E) protein [12], which has been shown to cluster into cellular membranes forming a pore, and the accessory protein Opern Reading Frame 3a (Orf3a), for which the evidence is less consistent [13].

These viroporins insert into the membranes of the endoplasmic reticulum, Golgi apparatus, and other intracellular compartments, disrupting ionic homeostasis and facilitating the assembly and release of new virions. Historically, the study of such viral proteins gained significant momentum with the M2 protein of influenza A virus (IAV), identified by Pinto et al. in the early 1990s [14], as one of the first definitively characterized virus-encoded ion channels, functioning specifically as a proton conduit. Importantly, this mechanistic understanding came after the development of amantadine, which was later recognized and clinically approved as the first antiviral drug targeting a viral ion channel, establishing a precedent for the druggability of such proteins [14]. Viral proteins with membrane-modifying or channel-related activity include HIV-1 Vpu [15] and IAV PB1-F2 [16]. The SARS-CoV-2 E protein, that is further reviewed here, is capable of transporting ions and is linked to pathological inflammatory responses aiding viral pathogenicity by inducing cellular stress responses, including ER stress, modulating autophagy, and activating the inflammasome [17]. However, classifying a viral protein such as E or Orf3a as a true ion channel rather than a general viroporin requires several experimental criteria to be met. These criteria include robust transmembrane conductance after heterologous expression, detection of single-channel current signatures, pharmacological sensitivity, measurable ion selectivity, and predictable functional effects of targeted mutations, particularly within transmembrane domains. This is an attempt to avoid weak classification, which leads to inadequately supported mechanistic claims and drug logic. Viroporins play a pivotal role in the dissemination of the virus within the host. As viral replication proceeds, the E protein and Orf3a protein are synthesized and incorporated into newly formed virions, enhancing their infectivity and virulence. The viroporins not only manipulate the host cell ion homeostasis but also facilitate immune evasion by altering the host cell’s intrinsic defense mechanisms [18,19], potentially contributing to mechanisms underpinning viral persistence. These processes underscore the critical importance of viroporins in the SARS-CoV-2 life cycle, making them compelling targets for therapeutic intervention.

A persistent challenge in the field is that the pathogenic importance of SARS-CoV-2 viroporins is widely recognized, whereas the extent to which individual proteins such as E and Orf3a meet the stricter functional definition of bona fide ion channels remains unresolved. This distinction is important because it affects both experimental interpretation and therapeutic targeting. We therefore treat E as the stronger case and Orf3a as a more unsettled one. The study of viroporins in the pathophysiology of SARS-CoV-2 offers significant insights into the viral life cycle and mechanisms of pathogenicity. Understanding how these viral proteins modulate host cell environments to favor viral replication, and immune evasion can lead to the development of targeted therapies that disrupt these processes, potentially reducing viral load and pathogenicity. In this review, we will examine the molecular basis of SARS-CoV-2 infection, with emphasis on viroporins, including the Envelope (E) protein and Orf3a. We also assess how these SARS-CoV-2 proteins are characterized as specific ion channels, while also recognizing their established importance as membrane-modifying viroporins indispensable for viral propagation and pathogenesis. Furthermore, their potential contribution to mechanisms underpinning viral persistence and immune evasion strategies will be discussed, highlighting them as compelling therapeutic targets to improve clinical outcomes in COVID-19.

## 2. SARS-CoV-2 Proteins and Their Functions

### 2.1. Replicative/Non-Structural Proteins

SARS-CoV-2 encodes a range of proteins categorized into replicative/non-structural, structural, and accessory/unique proteins. The replicative/non-structural proteins primarily facilitate the replication and transcription of viral RNA [5]. These proteins orchestrate the formation of the replication-transcription complex (RTC), essential for viral RNA synthesis. For instance, nsp12 acts as the RNA-dependent RNA polymerase [5], while nsp3 and nsp5 function as proteases processing the viral polyprotein [18]. Among these, nsp3 contains domains implicated in post-translational modification including ubiquitination activity. We note this point because the modification has been shown, but its downstream effect on E protein stability or function is still unclear.

### 2.2. Structural Proteins

Structural proteins provide the essential framework for virion assembly and are crucial for its assembly and entry into host cells. The principal structural proteins include the Spike (S) protein, which mediates virus entry into host cells by binding to the ACE2 (angiotensin-converting enzyme 2) and/or NRP1 (neuropilin) receptor [2], and the Envelope (E) protein, which is a major focus of this review. This small, hydrophobic transmembrane protein oligomerizes within membranes and clearly fits the definition of a viroporin. It is a minor structural protein involved in viral assembly, release, and pathogenesis [17]. Current evidence supports its localization to the ER-Golgi intermediate compartment (ERGIC) [20], and the function as a cation-selective ion channel [21]. Isolated amplification implies that it is integral to facilite virus production, packaging, and egress [19]. The Membrane (M) protein shapes the viral envelope and coordinates viral assembly [22], while the Nucleocapsid (N) protein binds to the viral RNA genome, forming the nucleocapsid structure involved in RNA replication and packaging [23].

### 2.3. Accessory/Unique Proteins

Accessory/unique proteins, though not essential for viral replication, assist the virus in evading host immune responses and augment its pathogenicity. Examples include the Orf3a protein, which functions as a viroporin with ion channel-like properties that may differ from those of the E protein in selectivity or regulation. We would describe Orf3a more cautiously than E, because the literature supports membrane activity more consistently than channel behavior. Its contribution to viral pathogenesis is probably due to the impact of viroporin activity on cell viability, in addition to the role in immune evasion [13,24]. Conversely, other accessory proteins, exemplified by Orf9b, have been shown to directly engage with mitochondria, influencing cellular processes including innate immune signaling, thereby illustrating the range of host cell pathways altered by SARS-CoV-2. Additional accessory proteins such as Orf6, Orf7a, Orf7b, Orf8, and Orf10 also modulate host immune responses and enhance viral replication [24,25].

## 3. Mechanisms of Viroporin Function

Understanding the mechanisms by which viroporins function is crucial for developing targeted therapies against viral infections. Viroporins are small viral transmembrane proteins that form ion channels or pores in host cell membranes. They are integral to various stages of the viral life cycle, including virus entry, intracellular trafficking, assembly, and release [7]. In the context of SARS-CoV-2, viroporins such as the envelope (E) protein and Orf3a play a critical role in modulating the host cell environment to facilitate viral replication and pathogenesis [18], and potentially contribute to the post-acute sequelae of SARS-CoV-2, also known as long-COVID syndrome [26,27]. Similar mechanisms have been observed in other viruses, such as HIV [15], influenza [14], hepatitis C [28], hepatitis A [29], Semliki Forest virus [30], poliovirus [31], rotavirus [32], and enterovirus [33].

At the mechanistic level, we see the central issue is not whether these proteins disturb membranes, as they have been shown to do so. The unresolved question is how the membrane effects produce the cell stress, inflammatory signaling, and tissue injury seen in disease. This section examines how viroporin-mediated alterations in membrane permeability, complemented by the specific ion channel activities inherent to proteins like E and Orf3a facilitate viral replication and dissemination. The disruption of cellular ion gradients (e.g., calcium and hydrogen ions) likely creates intracellular conditions favorable for viral enzymatic activity or assembly. Across systems, the evidence for E appears stronger and easier to reproduce than the evidence for Orf3a. An assessment of the E protein against established ion channel criteria reveals partial support. Electrophysiological investigations have confirmed membrane conductance induction and cation selectivity, while targeted mutagenesis within its transmembrane domain has affirmed impacts on ion transport and viral functions. What still seems less settled is the full single-channel profile and defined gating behavior, especially when compared with influenza A M2. Additionally, although certain compounds exhibit inhibitory effects, the identification of highly specific and potent pharmacological modulators comparable to Amantadine for M2 remains an ongoing endeavor. Therefore, overall, E protein is best supported as a viroporin with cation-conducting activity, although some channel properties remain incompletely defined.

Its documented channel activity perturbs calcium homeostasis, which is postulated to subsequently precipitate ER stress and potentially contribute to inflammasome activation pathways (discussed further in Section 7.2). Perturbation of calcium homeostasis emanating from E protein activity within the ER/Golgi network might exert indirect consequences on other organelles, potentially impacting mitochondrial function through calcium overload phenomena, although direct E protein interaction with mitochondria lacks robust empirical support. Furthermore, the modification of cellular homeostasis mechanisms such as autophagy by viroporin activity may promote viral maturation or facilitate immune evasion, collectively contributing to efficient viral production and egress.

Both enveloped and non-enveloped viruses exploit extracellular vesicles (EVs) as a strategy to broaden their tropism without immediate immune recognition [34]. As we advance our understanding of the viroporins of SARS-CoV-2, it is essential to consider their broader implications beyond the primary sites of infection. Viroporins’ ability to disrupt ion homeostasis and compromise membrane integrity effectively extends the viral impact beyond target cells, contributing to widespread tissue pathology and severe disease outcomes. This helps explain why tissue injury sometimes extends beyond cells with obvious receptor susceptibility.

### 3.1. Viroporin Impact on Non-Target Cells and Mechanisms of Spread

#### 3.1.1. Pathological Effects on Non-Target Cells

**Figure 1:**
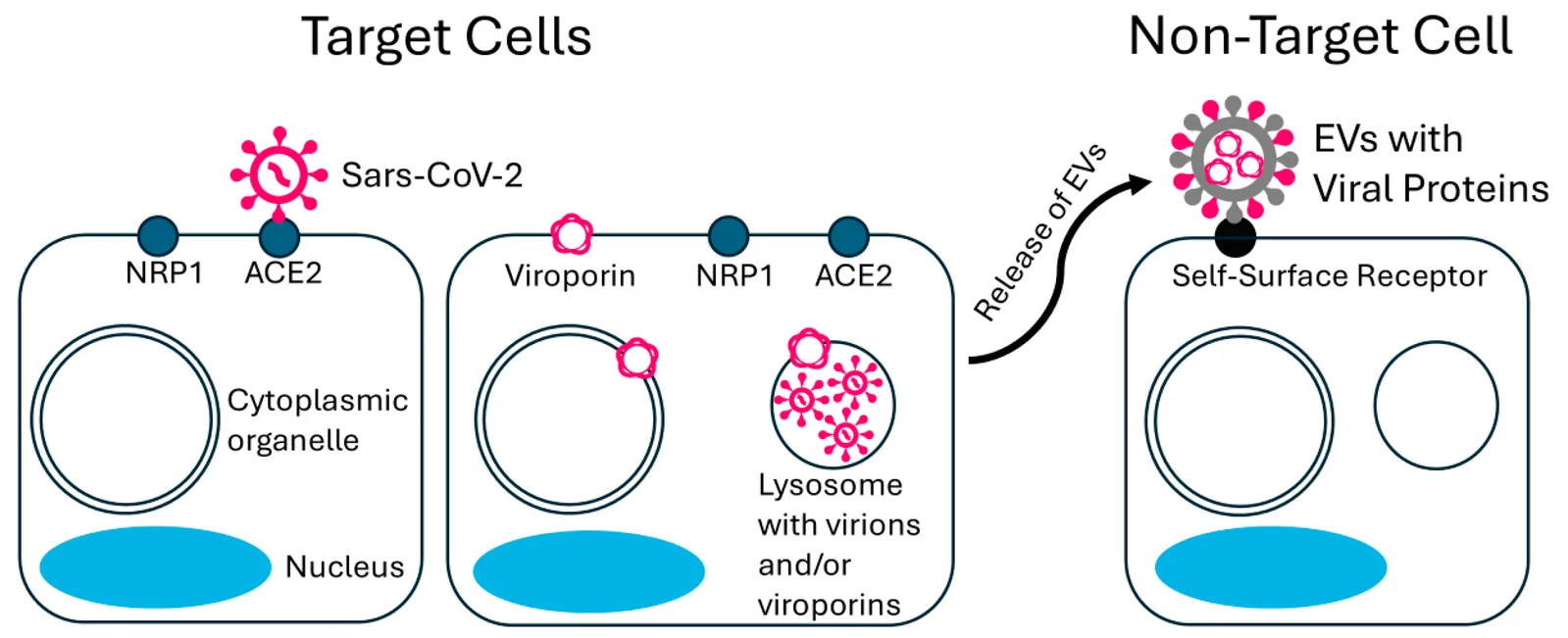
Viroporin dissemination and its impact on non-target cells through extracellular vesicles (EVs) in SARS-CoV-2 infection: mechanisms of viroporin dissemination and its impact on non-target cells via EVs in SARS-CoV-2 infection. The left panel shows target cells with NRP1 and/or ACE2 receptors facilitating viral entry and viroporin formation. The right panel illustrates how EVs, containing viroporins from infected cells, travel to and incorporate into non-target cells, contributing to widespread tissue pathology and viral dissemination.

Cells lacking ACE2 and/or NRP receptors can still be pathologically affected by viroporins like the SARS-CoV-2 E protein (Figure 1). In addition to the apoptotic mimicry of whole virions [19], the viroporins can integrate into neighboring cell membranes through EVs [19,35,36]. The integration of viroporins alone may be sufficient to lead to cell death [37]. We see this as a plausible route for damage in tissues with low ACE2 or NRP1 expression.

The release of coronavirus E protein in membrane vesicles from virus-infected cells and E protein-expressing cells has been documented [35], suggesting that viroporins can be packaged into EVs and spread to non-infected cells. Such activities contribute to broader viral dissemination and pathology beyond the initial sites of infection.

#### 3.1.2. Priming of Lipid Rafts by Extracellular Vesicles

**Figure 2:**
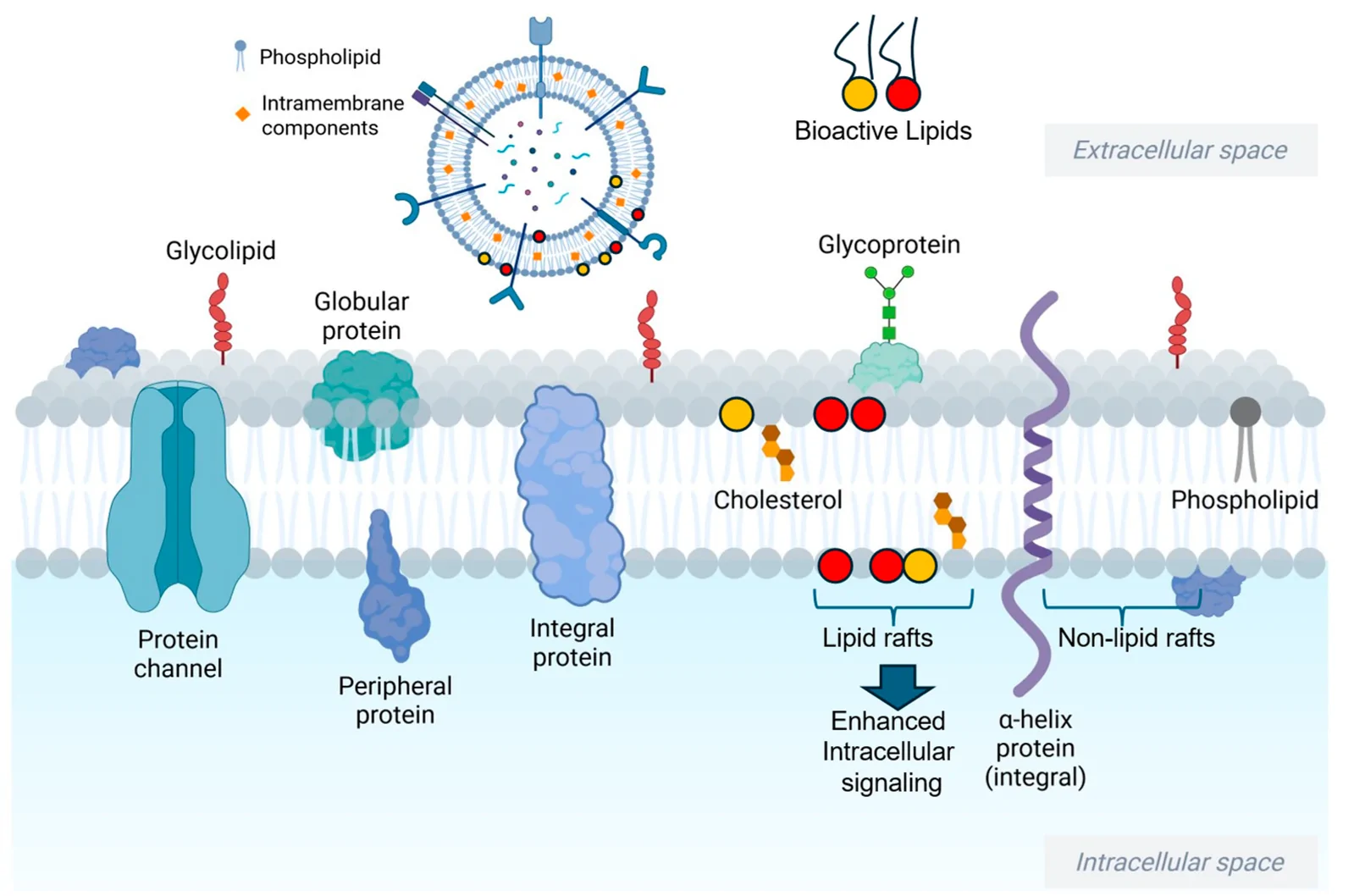
Viral entry mediated by bioactive lipids enriched in extracellular vesicles. Cellular uptake of extracellular vesicles released from infected cells after SARS-CoV-2 infection may lead to assembly and increased density of lipid rafts in healthy cells. In addition, the incorporation of extracellular vesicles into the plasma membrane of host cells may cause changes in membrane fluidity and permeability leading to viral particles entering the cells. Figure created in BioRender.com/AAA(2026).

A previous study by Darwish et al. reported that the uptake of extracellular vesicles enriched in bioactive lipids by small airway epithelial cells primes lipid rafts for the activation of intracellular signaling pathways [38]. Although this mechanism is not yet directly linked to SARS-CoV-2 viroporins, we are in favor of it being relevant with the possibility of infected-cell EVs altering recipient-cell membranes before any full viral entry event occurs. This mechanism of lipid raft priming leading to the downstream activation of signaling pathways in host cells may be important in the progression of COVID-19 disease (Figure 2). Additional studies are needed to determine whether viroporins are involved in this process. Lipid raft therapy has been investigated as a novel therapy for reducing the abundance of host lipid rafts to mitigate viral entry. For example, Carter et al. reported that disruption of lipid rafts in macrophage membranes using multiple cholesterol disruption agents significantly inhibited HIV entry and these effects could be reversed after the addition of water-soluble cholesterol [39]. Similarly, Sviridov et al. suggested that lipid raft therapy can potentially be effective in treating COVID-19 [40].

#### 3.1.3. Mechanisms of Viroporin Dissemination and Fate

In addition to direct viral entry and replication in target cells as a means of disseminating viral particles and machinery, extracellular vesicles play a critical role in spreading these particles, especially viroporins, between cells. Infected cells release vesicles incorporating viroporins, which travel through bodily fluids and impact distant cells, broadening the virus’s pathological footprint [35,41,42,43]. Additional mechanisms include cell-cell spread through syncytia formation [44] and spread through viral migrasomes [45], which carry viral particles and host cell materials such as damaged organelles that can integrate into neighboring cells. For instance, extracellular vesicles containing SARS-CoV-2 proteins are associated with multi-organ dysfunction and worse outcomes in patients with severe COVID-19 [46]. Together, these findings support EV-mediated spread as a plausible contributor to severe systemic disease.

After integration into host cell membranes, E proteins form ion channels that alter the cellular ionic balance. Cellular mechanisms like autophagy and the ubiquitin-proteasome system (UPS) attempt to degrade these foreign proteins. However, regarding its cellular degradation, direct evidence supporting turnover via the canonical ubiquitin-proteasome system (UPS) is currently scarce. During viral infection and associated stress, these pathways may be overwhelmed, allowing viroporins to persist and continue their pathogenic activity. We suspect this persistence plays a key role, especially in stressed tissues, because prolonged membrane disturbance would be enough to sustain inflammatory signaling even after active replication falls. This sustained presence is a probable factor contributing to prolonged ionic imbalance and chronic cellular stress, leading to long-term tissue damage and dysfunction. Ultimately, the interplay between direct viral and extracellular vesicle-mediated spread and the persistence of viral components like the E protein creates a complex pathogenic landscape in COVID-19.

## 4. Viroporin-Mediated Disruption of Lysosomal Function and Vaccine Efficacy

Lysosomes play a fundamental role in the cellular immune response, particularly in the context of vaccine efficacy. Their acidic environment is required for proteolytic enzymes such as cathepsins to degrade antigenic material into peptides suitable for MHC loading and presentation to CD4+ T helper cells [47].

However, viroporins disrupt lysosomal function. The E protein forms ion channels that compromise the lysosomal acidic environment by altering ion influx and efflux. This ion homeostasis disruption impairs the acidity-dependent proteolytic enzyme activity within the lysosomes, leading to suboptimal degradation of pathogenic components, including those of viral origin. The viral controlled neutralization of the lysosomal acidic environment by viroporins can weaken the immune response, making it harder for the body to effectively combat the virus [48,49]. Inhibiting viroporin activity may therefore help preserve lysosomal function, which is important for effective immune responses [50].

Moreover, the E protein impacts cellular ion channels through complex signaling pathways. For example, it serves as a ligand for Toll-like receptors (TLRs), activating pathways such as the c-Jun N-terminal kinase (JNK) pathway. This can lead to the upregulation of phosphodiesterase 4D (PDE4D), which decreases intracellular cyclic adenosine monophosphate (cAMP) levels by increased degradation [51]. Since cAMP is crucial for signal transduction and regulating various genes, including ion channels [52], its reduction can suppress cellular excitability.

Practically, if viroporin activity weakens lysosomal acidification, antigen processing suffers. If antigen processing suffers, host defense and vaccine response may both lose efficiency.

## 5. Viral Persistence in Different Tissues and Viroporin Impact

Viral persistence across tissues provides another context in which viroporin activity may be relevant. Viroporins, through ionic disruptions, may create conditions that favor viral persistence and reactivation. This concept applies not only to well-known latent viruses like herpesviruses (HSV and VZV) [53], HIV [54], and CMV [55] but also suggests a potential persistence mechanism for SARS-CoV-2 [56].

### Persistence Potential of SARS-CoV-2

Emerging evidence suggests SARS-CoV-2 can persist post-infection. Studies reviewed by McMillan et al. [57] as well as Serapide et al. [58] have shown SARS-CoV-2 RNA and/or protein can persist in tissues post-infection, suggesting potential long-term persistence rather than classical latency. Viroporins such as the E protein modify intracellular ionic conditions, potentially facilitating a persistence state. By disrupting calcium and proton homeostasis, viroporins might contribute to the evasion of host cell defenses that typically eliminate viral reservoirs. In compromised immune contexts, these ionic disruptions could enable virus components to persist undetected [57,58].

There are multiple studies demonstrating SARS-CoV-2 persistence in tissues including the lungs, gastrointestinal tract, and lymphoid organs long after the resolution of acute infection. This persistence may reflect immune evasion, and, in some settings, a cellular environment shaped by viroporin activity. Interactions with host proteins such as Protein Associated with Lin Seven 1 (PALS1) and Zonula Occludens-1 (ZO-1) disrupt cell junctions and may enhance viral retention and immune evasion [51].

The implications of SARS-CoV-2 persistence are significant, potentially contributing to long-COVID symptoms or enabling reactivation under certain conditions. Conditions of immunosuppression or stressors could theoretically trigger reactivation, cause recurrent symptoms or exacerbate disease progression. We would define persistence here narrowly, as prolonged presence of viral material or low-level replication, not classical latency. Understanding these mechanisms is critical for developing strategies to manage long-term effects and potential recurrent infections linked to COVID-19. The ongoing study of viroporin activity in SARS-CoV-2 may help guide strategies to counteract its persistence and potential reactivation. Clarifying the contribution of viroporins to viral persistence—defined here as the prolonged presence of viral material or low-level replication, rather than classical latency—may also inform efforts to reduce post-acute sequelae.

## 6. Role of Protein–Protein Interactions in Viral Pathogenesis

The SARS-CoV-2 envelope (E) protein has an important role in viral replication, pathogenesis, and host cell interaction, making it a significant therapeutic target. Its functions include virion assembly and release, activation of the inflammasome, and disruption of cellular ion channels as a viroporin. The E protein’s interaction with host and viral proteins further complicates the disease’s progression and severity.

### 6.1. Functional Interactions

Guarnieri et al. [59] and Nieto-Torres [60] have previously shown that the E protein’s interactions go beyond traditional viral assembly roles. It forms ion channels contributing to cellular ion homeostasis disruption, leading to inflammasome activation and the immunopathological consequences observed in COVID-19 [59,60]. The C-terminal domain, containing a PDZ-binding motif (PBM), interacts with host cell junction proteins such as PALS1, ZO-1, and syntenin, affecting cell polarity and potentially leading to increased viral entry and inflammation [51,61,62].

Duart et al. compared E proteins in SARS-CoV-2 with six other human coronaviruses and showed an overall 94% amino acid sequence similarity of E proteins between SARS-CoV-1 and SARS-CoV-2, with continued closeness in membrane topology analysis [63]. As mentioned earlier, while the fate of E proteins is yet to be determined in SARS-CoV-2, it is plausible that similar mechanisms occur. For example, N-terminal ubiquitin-like domain-1 of SARS-CoV-1 non-structural protein 3 (nsp3) mediates contact and ubiquitination of the E protein in infected cells [64]. Furthermore, the similarities include interactions with host proteins such as PALS1 and ZO-1 that enhance the E protein’s pathogenicity by disrupting cell junctions and increasing permeability, which may allow noxious agents into infected tissues, thereby exacerbating inflammation [65].

### 6.2. Ionic Channel Dysfunction

The E protein’s ion channel activity affects several ion channels, impacting cellular function. Viroporin activity forms cation channels impacting sodium (Na^+^) and calcium (Ca^2+^) levels. Increased intracellular sodium can depolarize the cell membrane and disrupt excitability and signaling pathways, contributing to neurological symptoms in COVID-19 patients, such as anosmia and ageusia. Similarly, disruptions in calcium homeostasis, primarily through the E protein, elevate intracellular calcium levels, activating inflammatory and apoptotic pathways, possibly contributing to muscle fatigue and myalgia [66].

In addition to Na^+^ and Ca^2+^ channels, chloride (Cl^−^) and potassium (K^+^) channels are affected. In epithelial cells, the E protein activates both TLR2/4 receptors and the JNK signaling pathway. This ultimately leads to an increase in intracellular Cl^−^ concentration due to the upregulation of PDE4D expression, potentially underlying respiratory and gastrointestinal symptoms of COVID-19. Disruptions in potassium ion balance, although less studied, are involved in cardiac complications including arrhythmias. Moreover, the PBM motif of the E protein affects the expression of host genes involved in ion transport and homeostasis [37,51].

One clinically relevant consequence of SARS-CoV-2, particularly through its envelope (E) protein, is its effect on the epithelial sodium channel (ENaC). The SARS-CoV-2 spike (S) protein contains a furin-like cleavage site at the S1/S2 junction, which mimics the cleavage site in ENaC, a critical regulator of sodium and fluid transport in epithelial cells [37,67]. The cleavage by furin or furin-like proteases normally activates ENaC, facilitating sodium uptake and subsequent water reabsorption across the cell membrane. However, the competitive interaction of the SARS-CoV-2 spike protein with these proteases may lead to inefficient cleavage and activation of ENaC. This interference results in reduced sodium ion reabsorption through the channel, significantly compromising fluid clearance from the alveolar spaces. The dampened function of ENaC due to suboptimal activation could translate into a higher risk of pulmonary edema, as fluid accumulates in the lungs, leading to the severe respiratory distress observed in COVID-19 patients [67].

Moreover, the viroporin activity of the SARS-CoV-2 E protein contributes further to the disruption of ENaC expression and function by destabilizing intracellular ionic homeostasis. Viroporins, by increasing intracellular calcium levels, significantly impact various cellular processes. Elevated intracellular calcium can activate protein kinase C (PKC), which has been shown to downregulate ENaC surface expression by promoting retrieval of the channel from the plasma membrane and its degradation. Furthermore, the sustained rise in intracellular calcium mediated by viroporin channels can inhibit ENaC transcriptionally and post-translationally, further reducing its activity [67]. Together, these effects may contribute to fluid accumulation in the airways, reduced oxygen exchange, and severe hypoxemia. We can speculate that ENaC dysregulation during SARS-CoV-2 infection involves protease competition, imbalance between protease and protease inhibitor levels, and alveolar fluid handling. 

The hyperinflammatory response characteristic of acute respiratory distress syndrome in COVID-19 is partially driven by the E protein. It induces high levels of inflammatory cytokines including IL-1β and IL-6, contributing to the cytokine storm. The interaction between the E protein and TLR2 triggers NF-κB activation, resulting in pro-inflammatory chemokine production. These findings propose that the E protein is more than a passive structural protein and it actively shapes the inflammatory state of infected tissue. Inhibition of the E protein’s ion channel activity reduces cytokine production, highlighting its role in immunopathology [37,60,66].

## 7. Impact of Viroporins on Mitochondrial Function and Pathogenesis

Mitochondria, known for their role in energy production, regulation of metabolic pathways, and apoptosis, are significantly affected by viroporin activity. Viroporins like the SARS-CoV-2 E protein can profoundly disrupt mitochondrial functions, leading to a cascade of pathophysiological consequences that amplify viral pathogenicity. However, direct evidence for E protein interaction with mitochondria remains limited, and many of the observed effects are likely indirect consequences of altered cellular ion homeostasis, particularly calcium dysregulation.

Viroporins (or the downstream effect of their activity) disrupt mitochondrial membrane potential, impairing the electron transport chain (ETC) and reducing ATP production, directly affecting cellular energy state and overall cell function. This disruption also elevates the production of reactive oxygen species (ROS), leading to oxidative stress and damage to mitochondrial DNA (mtDNA), proteins, and lipids. Damaged mtDNA can act as danger-associated molecular patterns (DAMPs), triggering innate immune responses such as the activation of the NLRP3 inflammasome, further driving inflammation and tissue damage [59,68]. Viroporin-mediated (or induced) calcium influx into mitochondria elevates mitochondrial reactive oxygen species (mROS) production, potentially releasing fragmented mtDNA through the mitochondrial permeability transition pore (mtPTP). These events contribute to COVID-19 pathophysiology by affecting mitochondrial function and energy production [59,68]. Viroporin activity also affects mitochondrial dynamics, impacting processes of fission and fusion vital for maintaining mitochondrial integrity, distribution, and metabolic response. It impairs mitophagy, leading to dysfunctional mitochondria accumulation and exacerbating cellular stress and damage.

In addition to oxidative stress and dynamics disruption, viroporins influence apoptosis by inducing mitochondrial outer membrane permeabilization (MOMP), releasing pro-apoptotic factors like cytochrome c into the cytoplasm and triggering the caspase cascade. This results in increased cell death, tissue damage, and disease progression. Chronic apoptotic pathway activation depletes functional cells and impairs tissue regeneration and repair. It remains that the strongest version of this argument is indirect. E protein alters calcium handling in the ER-Golgi network, and mitochondria are also adversely affected.

### 7.1. SARS-CoV-2 Viroporin Interaction with Immune Pathways

Mitochondrial disruption caused by viroporins extends to immune system interactions. For example, the release of mtDNA into the cytoplasm activates the Cyclic GMP-AMP Synthase—STimulator of INterferon Genes (cGAS-STING) signaling pathway, which increases the production of type I interferons and pro-inflammatory cytokines. Additionally, excessive ROS produced by dysfunctional mitochondria modulate immune cell activity, contributing to chronic inflammation and potential autoimmune responses [68].

Furthermore, viroporin activity affects signaling pathways related to other ion channels. The E protein’s competition for protein-sorting machinery and the resulting ionic gradient dysregulation lead to downstream consequences. The E protein can act as a ligand for TLRs, such as TLR2, activating pathways like JNK signaling. This activation can upregulate PDE4D, decreasing intracellular cyclic adenosine monophosphate (cAMP) levels through increased degradation [51]. Since cAMP is a crucial regulator of various genes, including ion channels [52], its reduction suppresses cellular excitability [69,70,71]. It stands to reason how ionic disturbance and inflammatory signaling appear together in the same pathway, rather than as separate effects.

### 7.2. SARS-CoV-2 Viroporin-Mediated Activation of the NLRP3-Inflammasome

**Figure 3:**
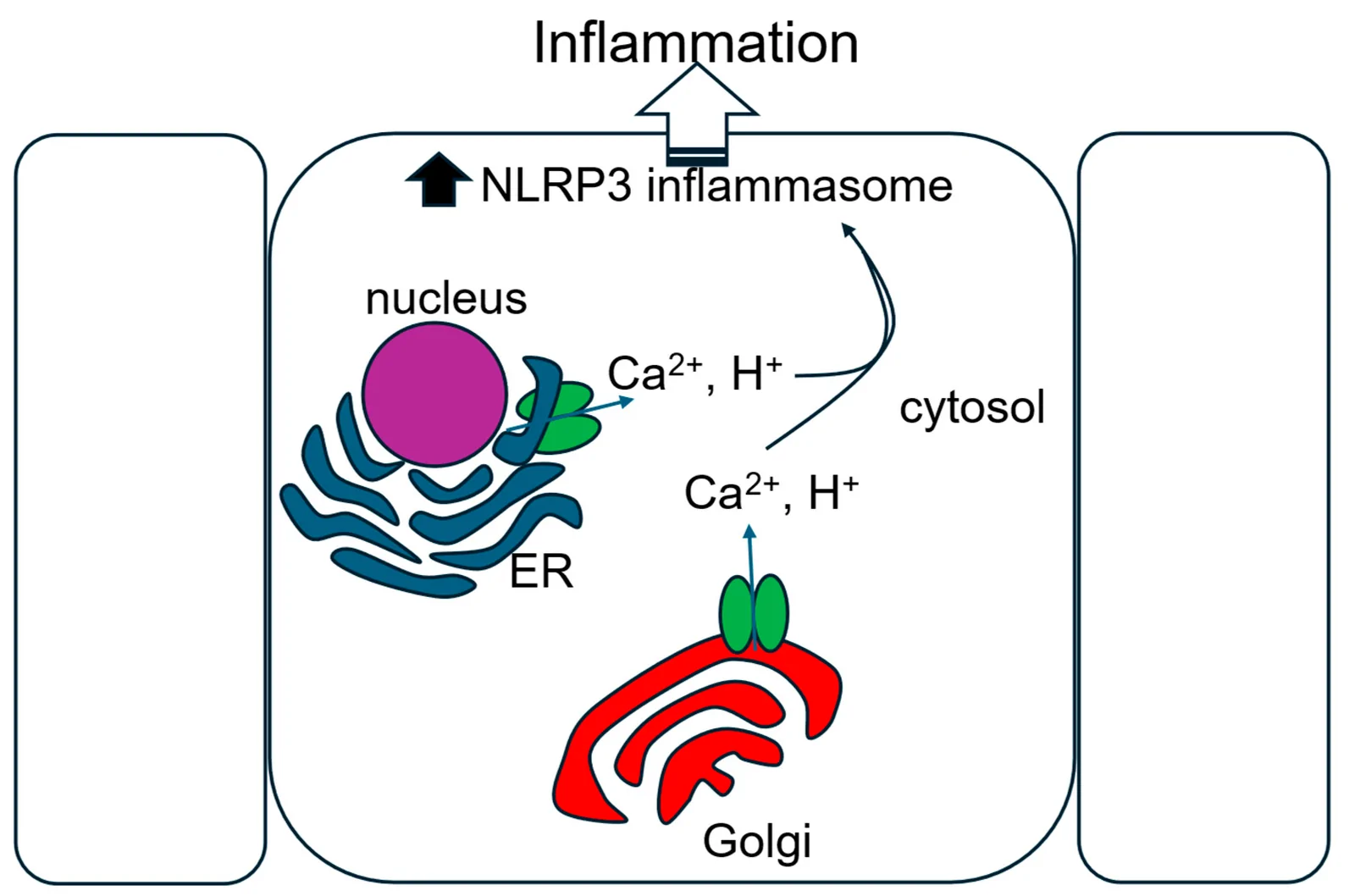
Viroporin channel activity-mediated activation of the NLRP3 inflammasome leading to inflammation. Viroporin enrichment in intracellular host cell organelles including the endoplasmic reticulum (ER) and Golgi apparatus leads to the movement of calcium and hydrogen ions into the cytoplasmic space. The ionic imbalance leads to the assembly and activation of the NLRP3 inflammasome and consequently, inflammation. Solid black arrow indicates increased activation.

The enrichment of viroporins in intracellular host organelles including the endoplasmic reticulum and Golgi apparatus after viral infection is thought to contribute to the movement of calcium and hydrogen ions into the cytoplasm, thereby promoting the assembly and activation of the NLRP3 inflammasome (Figure 3). The SARS-CoV-2 viroporin protein Orf3a was reported to increase cytoplasmic levels of calcium, and the elevated mitochondrial calcium was shown to increase reactive oxygen species levels in mitochondria and induce the release of mtDNA into the cytosol, leading to the activation of the NLRP3 inflammasome [59]. In another study, the SARS-CoV-2 viroporins ORF3a, E protein and M protein were reported to be capable of activating the inflammasome in human macrophages, leading to the release of IL-1 from endothelial and epithelial cells [72]. A study by Nieto-Torres et al. showed that IL-1β and inflammasome activation were reduced in lung airways of mice infected with viruses lacking a functional E protein [60]. Taken together, these studies suggest that multiple viroporins are involved in inflammasome activation. This warns against treating the E protein as the only inflammatory driver. The network looks redundant and additional studies are necessary to further investigate whether the absence of one viroporin can be compensated by the upregulation of another viroporin.

## 8. Decreased Viroporin Activity and Pathogenicity

Reduced viroporin activity is generally associated with lower viral fitness and pathogenicity. When viruses possess mutations that reduce viroporin function, their ability to replicate and cause harm is often diminished. Multiple studies support this, showing that alterations can lead to impaired viral replication, decreased virulence, and an overall reduction in pathogenicity [60,61].

The comparative genomic analysis done by Rizwan et al. showed that the SARS-CoV-2 variant Beta (B.1.351) had a C212U/Pro71Leu mutation in the E protein. This mutation was statistically associated with severe morbidity and a high mortality rate [73]. Liu et al. showed that all SARS-CoV-2 variants have pan-human coronavirus-conserved nucleotide positions in the E gene with superimposed conservation within SARS-CoV-2 variants. The E mutations in variants of concern were associated with disease severity as well as milder clinical presentation [74].

Even in SARS-CoV-1, whose E protein is now considered a representation of the one seen in SARS-CoV-2, inducing a mutation in the E protein resulted in diminished ability to disrupt ionic homeostasis, leading to less production of the inflammatory trigger interleukin-1β. The wildtype E protein with intact ion channel activity had interleukin-1β overproduction, negatively impacting cell viability [60].

Reduced viroporin function also affects the virus’s ability to evade immune detection. Studies indicate that SARS-CoV-2 strains with E protein mutations are less capable of evading immune responses, resulting in decreased virulence [75].

Despite these findings, some coronaviruses exhibit resilience through compensatory mechanisms. For instance, the Orf3a protein in SARS-CoV-1 can partly substitute for E protein functions, maintaining some level of pathogenicity even when E protein activity is compromised. This highlights the complex interplay of viral proteins and necessitates a comprehensive approach when targeting viroporin functions in therapeutic interventions [58]. This is one of the clearest arguments against single-target optimism. If the function of one viroporin is attenuated and another compensates for its loss, drug response will be more difficult to predict. Addressing potential functional compensation among different viral viroporins is therefore a key challenge for therapeutic development.

Overall, available data are consistent with a link between reduced viroporin function and reduced pathogenicity. This insight has significant implications for therapeutic strategies. By targeting viroporin activity, it may be possible to impair the virus’s ability to manipulate host cell environments, resulting in less virulent infections and improved clinical outcomes. Prioritization should given to inhibitors against the best-supported and most reproducible activities first, especially those tied to calcium dysregulation, inflammasome activation, and viral egress. This approach aligns well with developing viroporin inhibitors, which hold significant promise as potential antiviral treatments. These inhibitors could render viroporins non-functional, disrupting the viral lifecycle at multiple stages and reducing the severity of infections like COVID-19, ultimately enhancing patient care and management in viral diseases.

## 9. Conclusions

The study of ion channels, particularly viroporins, in the pathophysiology of SARS-CoV-2 has revealed significant insights into the viral life cycle and mechanisms of pathogenicity. After reviewing this literature, we think the most defensible claim is modest but clear. SARS-CoV-2 viroporins disturb host cell ion balance, drive cell stress, and contribute to pathogenesis. This review consolidates evidence highlighting the pivotal role of viral proteins such as the E protein and Orf3a acting primarily through the disruption of host ionic homeostasis. Viroporins such as the E protein play multifaceted roles in disrupting host cell ionic homeostasis, manipulating cellular processes like ER stress and autophagy, and contributing to viral persistence and immune evasion. The case for E is stronger. The case for Orf3a is less stable across systems and methods.

Taken together, the evidence supports a consistent view: SARS-CoV-2 viroporins are important drivers of host cell dysregulation and pathogenesis, even as key questions remain about whether all proposed activities should be classified as canonical ion channel behavior. At present, the case is strongest for E protein, whereas the evidence for Orf3a remains more variable across systems and experimental approaches.

The clearest next step is to define which viroporin activities are both reproducible and druggable. Sustained investigation into these viral proteins employing stringent biophysical and functional methodologies is crucial. It is important now to narrow the field to a smaller set of mechanisms with repeated experimental support, especially calcium dysregulation, inflammasome activation, and viral egress. Targeting viral protein functionality presents a distinct strategic avenue for mitigating viral replication and associated disease manifestations. Continued efforts will be essential to translating these findings into effective therapies for mitigating SARS-CoV-2 pathogenicity and improving patient outcomes.

Despite significant advances, critical research gaps persist in our understanding of SARS-CoV-2 viroporins. Future investigations should prioritize the complete biophysical characterization of proteins like E and Orf3a, including high-resolution structural data and detailed electrophysiological profiles, to definitively classify their ion channel properties. Elucidating the precise molecular mechanisms by which these viroporins modulate host cellular pathways, their specific roles in viral persistence and the development of post-acute sequelae such as long COVID, and their interplay in different cell types remains paramount. Furthermore, the development and rigorous testing of highly specific viroporin inhibitors are crucial, alongside strategies to address potential viral compensatory mechanisms. The big hope for the field is to produce more therapies that can withstand viral evolution. Addressing these gaps will not only deepen our comprehension of COVID-19 pathogenesis but also pave the way for novel therapeutic interventions targeting these viral vulnerabilities, potentially enhancing vaccine efficacy and improving patient outcomes against current and future coronavirus threats.

By preserving mitochondrial function, maintaining lysosomal integrity, and mitigating ionic imbalances, the potential for improved therapeutic interventions targeting viroporins could improve our approach to combating viral infections. The development of viroporin inhibitors, alongside existing antiviral strategies, represents a significant step forward. Progress depends on separating what has been shown repeatedly from what still rests on inference.

## Data Availability

No new data were created or analyzed in this study. Data sharing is not applicable to this article.

## Abbreviations

The following abbreviations are used in this manuscript:

ACE2	Angiotensin-Converting Enzyme 2
ARDS	Acute Respiratory Distress Syndrome
cAMP	Cyclic Adenosine Monophosphate
cGAS-STING	Cyclic GMP-AMP Synthase—STimulator of INterferon Genes
CFTR	Cystic Fibrosis Transmembrane Conductance Regulator
COVID-19	Coronavirus Disease 2019
DAMPs	Danger-Associated Molecular Patterns
ENaC	Epithelial Sodium Channel
E protein	Envelope Protein
ER	Endoplasmic Reticulum
EVs	Extracellular Vesicles
IL	Interleukin
JNK	c-Jun N-terminal Kinase
MHC	Major Histocompatibility Complex
M protein	Membrane Protein
MOMP	Mitochondrial Outer Membrane Permeabilization
mtDNA	Mitochondrial DNA
mtPTP	Mitochondrial Permeability Transition Pore
mROS	Mitochondrial Reactive Oxygen Species
nsp	Non-Structural Protein
NRP1	Neuropilin-1
Orf	Open Reading Frame
PBM	PDZ-Binding Motif
PDZ	Post-synaptic Density Protein, Drosophila Disc Large Tumor Suppressor, Zonula Occludens-1
PDE4D	Phosphodiesterase 4D
PALS1	Protein Associated with Lin Seven 1
pH	Potential of Hydrogen
PKC	Protein Kinase C
ROS	Reactive Oxygen Species
RTC	Replication-Transcription Complex
SARS-CoV	Severe Acute Respiratory Syndrome Coronavirus
SARS-CoV-2	Severe Acute Respiratory Syndrome Coronavirus 2
TLRs	Toll-Like Receptors
TMPRSS2	Transmembrane Protease, Serine 2
UPS	Ubiquitin-Proteasome System
ZO-1	Zonula Occludens-1
